# Co-Regulation of the DAF-16 Target Gene, *cyp-35B1/dod-13*, by HSF-1 in *C. elegans* Dauer Larvae and *daf-2* Insulin Pathway Mutants

**DOI:** 10.1371/journal.pone.0017369

**Published:** 2011-03-09

**Authors:** Wendy B. Iser, Mark A. Wilson, William H. Wood, Kevin Becker, Catherine A. Wolkow

**Affiliations:** 1 Invertebrate Molecular Genetics Unit, Laboratory of Neurosciences, Research Resources Branch, NIA Intramural Research Program, NIH Biomedical Research Center, Baltimore, Maryland, United States of America; 2 Gene Expression and Genomics Unit, Research Resources Branch, NIA Intramural Research Program, NIH Biomedical Research Center, Baltimore, Maryland, United States of America; University of Washington, United States of America

## Abstract

Insulin/IGF-I-like signaling (IIS) has both cell autonomous and non-autonomous functions. In some cases, targets through which IIS regulates cell-autonomous functions, such as cell growth and metabolism, have been identified. In contrast, targets for many non-autonomous IIS functions, such as *C. elegans* dauer morphogenesis, remain elusive. Here, we report the use of genomic and genetic approaches to identify potential non-autonomous targets of *C. elegans* IIS. First, we used transcriptional microarrays to identify target genes regulated non-autonomously by IIS in the intestine or in neurons. *C. elegans* IIS controls expression of a number of stress response genes, which were differentially regulated by tissue-restricted IIS. In particular, expression of *sod-3*, a MnSOD enzyme, was not regulated by tissue-restricted IIS on the microarrays, while expression of *hsp-16* genes was rescued back to wildtype by tissue restricted IIS. One IIS target regulated non-autonomously by *age-1* was *cyp-35B1/dod-13*, encoding a cytochrome P450. Genetic analysis of the *cyp-35B1* promoter showed both DAF-16 and HSF-1 are direct regulators. Based on these findings, we propose that *hsf-1* may participate in the pathways mediating non-autonomous activities of *age-1* in *C. elegans*.

## Introduction

Insulin/IGF-I-like signaling (IIS) is a highly conserved pathway for promoting growth under replete conditions. Growth control is cell-autonomously regulated by IIS to determine cell and organ size in the affected tissue [1], [2]. In addition to this well-conserved cell-autonomous function, IIS can also have non-autonomous effects on other parts of the body. These actions of IIS have been uncovered in *C. elegans*, *Drosophila* and mice, through studies of mosaic animals with IIS restricted to specific tissues. In worms and flies, tissue-restricted IIS non-autonomously regulates development and adult longevity [3], [4], [5], [6], [7], [8]. In mice, brain-specific insulin receptor deletion is associated with obesity and low fertility, likely reflecting hormonal disruptions [9]. Tissue-restricted IIS could confer these non-autonomous effects either specifically, through endocrine outputs, or non-specifically, through pleiotropic phenotypes resulting from tissue dysfunction due to inadequate growth. Thus, these findings raise new challenges for identifying the downstream pathways mediating non-autonomous effects of IIS [10].

This question can be investigated in *C. elegans*, for which the major IIS pathway components have been identified. These include *daf-2*, encoding the sole *C. elegans* insulin/IGF-I receptor-like protein, and *age-1*, encoding a p110 PI3K catalytic subunit that is the primary DAF-2/IR effector [11], [12], [13], [14]. The major downstream target of *daf-2* and *age-1* is *daf-16*, which encodes a FOXO transcription factor antagonized by DAF-2 signaling [15], [16]. In *C. elegans*, the *daf-2* pathway acts at both the cellular and organism level. At the cellular level, the *daf-2* pathway cell-autonomously regulates *sod-3* expression [6], [17], [18]. A second cell-autonomous output of the *daf-2* pathway is the regulation of FIRE response sensitivity in intestinal cells [8]. Two types of behavioral plasticity are also regulated cell-autonomously by *daf-2* [19]. The non-autonomous outputs of *daf-2* regulate organismal phenotypes. The *daf-2* pathway promotes reproductive development and prevents dauer larval arrest under replete conditions [14], [20], [21], [22]. In adult animals, the *daf-2* pathway promotes wildtype longevity and normal stress resistance [11], [23], [24], [25], [26], [27]. Both dauer arrest and adult longevity are controlled non-autonomously by *daf-2* and *age-1* activity from several cell types [3], [7], [8].

The downstream effectors for *daf-2* non-autonomous regulation of dauer arrest and adult longevity are not known. *daf-16*, the major cell-autonomous *daf-2* target, regulates longevity primarily from intestinal cells [6]. A working model proposes that *daf-2* activity can regulate *daf-16* through both the cell-autonomous pathway, via *age-1* and *akt-1*, and non-autonomously, through unidentified pathways [8]. The *daf-2* pathway's non-cell autonomous actions may reflect crosstalk with other signaling pathways that convergently regulate dauer arrest and adult longevity. One candidate is the heat-shock transcription factor, encoded by the *hsf-1* gene, which regulates lifespan, proteotoxicity and dauer arrest in collaboration with *daf-16* [16], [27], [28], [29]. HSFs are highly conserved and direct the expression of heat-shock proteins in response to thermal stress. In *C. elegans*, *hsf-1* also promotes the expression of other, non-*hsp*, targets in *daf-2* mutants through both *daf-16*-dependent and independent mechanisms [28].

To identify factors mediating the non-cell autonomous effects of the *daf-2* pathway upon dauer arrest, we searched for transcriptional targets regulated non-autonomously by *age-1* and then analyzed factors directing their regulation in response to the *daf-2/age-1* pathway. Using microarrays, we examined gene expression in animals with *age-1* activity restricted to neurons or gut, and the results were compared with gene expression in wildtype animals and zygotically null *age-1* mutants (*m+z-*). This approach identified a collection of transcripts that were potentially regulated by *age-1* in a non-cell autonomous fashion. We characterized the *cis-* and *trans-*requirements for *daf-2*-dependent expression of on of these non-autonomous targets, *cyp-35B1/dod-13*. The findings suggest that *hsf-1* may be a component of pathways mediating *age-1* non-autonomous activities.

## Results

### Gene expression patterns in animals with tissue-restricted *age-1* activity

**Figure 1:**
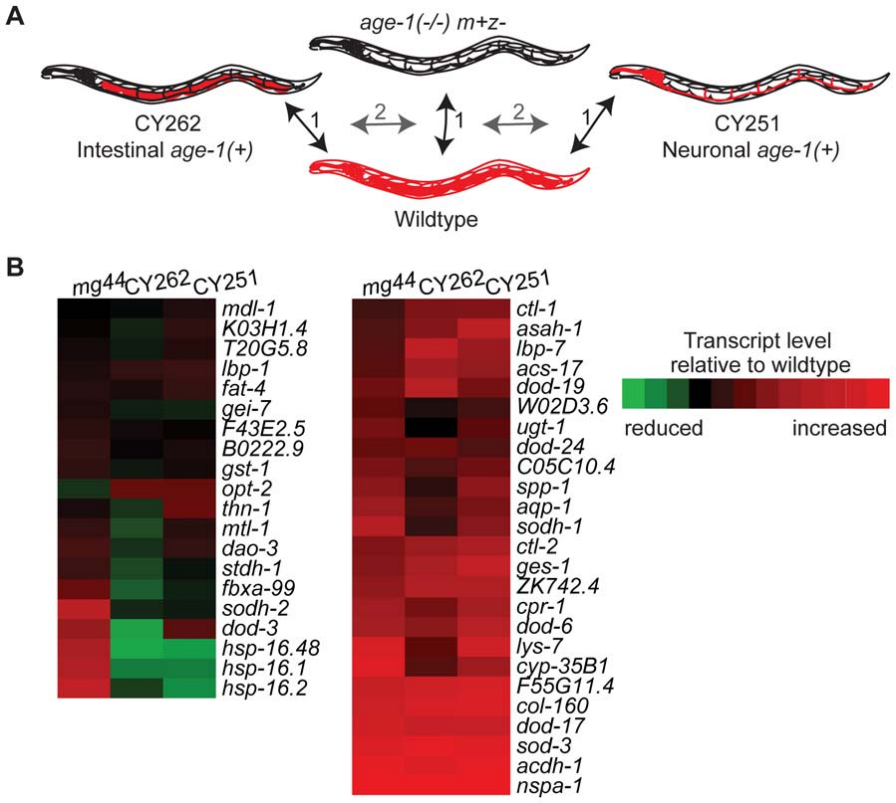
Transcriptional microarrays were used to identify non-autonomous *age-1* target genes. Global gene expression was compared in three strains; zygotic null *age-1(mg44)* adults, CY251 (*age-1(mg44)* with neuronally-restricted *age-1* expression) and CY262 (*age-1(mg44)* with intestinally-restricted *age-1* expression). All 3 strains were compared to the wildtype (*age-1(+)*). The dauer-constitutive phenotype of *age-1(mg44)* is maternal-effect. Therefore, this analysis used zygotic *age-1(mg44)* homozygotes (z-) from heterozygous *age-1(mg44/+)* parents (m+), which developed to adulthood due to maternal *age-1* activity. Tissues with *age-1* activity are colored orange; tissues lacking *age-1* activity are outlined in black. A. Strategy for analyzing microarray results to screen for potential non-autonomous *age-1* target genes. Step (1): Transcript abundance in all 3 strains was compared to wildtype. Step (2): Transcript abundance in CY262 and CY251 was compared to *age-1(mg44)*. Transcripts altered in *age-1(mg44)* (p<0.05 vs wildtype) and rescued in both CY262 and CY251 (p≥0.05 vs wildtype) were considered potential non-autonomous *age-1* targets. B. Heat map showing relative expression levels for 45 genes previously identified as *daf-2* targets [30]. The results show that elevated expression of 3 *hsp-16* genes in *age-1(mg44)* animals was rescued to low levels by tissue-restricted *age-1* activity, suggesting these may be non-autonomously regulated by *age-1*. However, *sod-3* levels remained elevated in the presence of tissue-restricted *age-1* activity, consistent with findings that the *daf-2* pathway cell-autonomously regulates *sod-3* in many tissues [6].

In order to search for targets regulated non-autonomously by *age-1*, gene expression was analyzed in animals with tissue-restricted *age-1* activity. This analysis compared gene expression in zygotically null *age-1* mutants (*age-1(mg44)*(m+z-)) with that in *age-1(mg44)* animals carrying transgenes directing neuronally-restricted (CY251) or intestinally-restricted (CY262) *age-1* expression (Fig. 1A). Both neuronal and intestinal *age-1* expression rescued constitutive dauer arrest of *age-1(mg44)* [8]. The extended lifespan of *age-1(mg44)* adults was also rescued by *age-1* expression in either tissue, although CY262 more strongly rescued adult longevity than CY251, consistent with a critical role for intestinal *daf-16* activity for extended lifespan [6].

Since *age-1* and *daf-2* mutants share many phenotypes, we expected that the *age-1* and *daf-2* transcriptomes would be similar. Therefore, we compared our results for *age-1(mg44)* and those of a previous study of gene expression in *daf-2* pathway mutants [30]. Of 113 *daf-2* targets which were also significantly changed in our experiment, 73% were changed concordantly in *age-1(mg44)* adults (fold-change p≤0.05, t-test) (Table S1). Considering the differences in reference pools and growth conditions, these results indicate high concordance of the *age-1* and *daf-2* transcriptomes, consistent with the fact that *age-1* and *daf-2* have similar mutant phenotypes [14]. These findings support the role of AGE-1/PI3K as the major effector for DAF-2 signaling.

The goal of this analysis was to identify *age-1* target genes that could be regulated non-autonomously by the *age-1* pathway. We reasoned that non-autonomous targets would be rescued to wildtype levels in the strains CY262 and CY251, which express *age-1* only in the intestine or neurons, respectively. Using these criteria, we examined the effect of tissue-restricted *age-1* activity on the 82 transcripts whose expression was regulated concordantly in *age-1* and *daf-2* mutants. We found that 30% (25 targets) were rescued in both CY262 and CY251, one target was rescued only in CY251, three targets were rescued in only CY262 and 65% (53 targets) were not rescued in either strain or had inconclusive data (Table S1). The targets whose expression was rescued in both CY251 and CY262 are potential non-autonomous *age-1* targets (Fig. 1B, Table S1). These included genes involved in a variety of processes, such as antimicrobial defense (*clec-13*, *lys-7*), reproduction (*vit-2*, *-4* and *-5*), catalysis (*F09F7.7* dioxygenase) and metabolism (*acr-2*). Little expression data was available for these targets, although two are reportedly intestinal (*lys-7*, *lipl-4*) and four have complex expression patterns (*cdr-2*, *F09F7.7*, *acs-2* and *F552H3.5*).

We then conducted this comparison for 791 transcripts that were significantly and reproducibly overrepresented in *age-1(mg44)* adults versus wildtype adults (at least 2-fold overrepresented, p≤0.05), without regard to their inclusion in the *daf-2* transcriptome (Table S2). We set two criteria for rescue of *age-1* targets in these strains. First, *age-1* targets were defined as being more than 2-fold overexpressed in comparison with wildtype levels with a p-value<0.05 (t-test vs wildtype). For rescue in CY262 or CY251, expression was both insignificantly different from that in wildtype animals (p≥0.05) and significantly different from that in *age-1(mg44)* animals (p<0.05). Within the group of 791 *age-1*-upregulated transcripts, 127 (16%) were rescued in both CY262 and CY251, and were potentially regulated non-autonomously by *age-1*. This group was composed of genes involved in a variety of biological processes, including defense or signaling (7 glutathione S-transferases, 5 cytochrome P450s, 6 lectins, 3 alcohol dehydrogenases, 4 glucuronosyltransferase and 3 nuclear hormone receptors). We expect that some non-autonomous *age-1* targets should be expressed outside of the *age-1*-expressing tissues in CY262 and CY251. Therefore, we surveyed the available expression data for these genes. Of the 127 potentially non-autonomous *age-1* targets, expression data was available for 18 (Wormbase, release 190). Eleven genes were reportedly expressed in the intestine, with 5 exclusively intestinal, although the significance of intestinal expression is unclear since this is a common site for promiscuous transgene expression in *C. elegans* [8]. The targets that were not exclusively intestinal were expressed in a variety of tissues, including neurons (7), hypodermis (8), the gonad (2), epidermal seam cells (3), muscle (5) and the pharynx (5). This finding is consistent with the idea that *age-1* non-autonomous outputs might target many of the body's tissues.

Using these rescue criteria, we also found that 12 targets were preferentially rescued in CY251 animals, while 37 were preferentially rescued in CY262 animals. The targets rescued preferentially in CY251 included one metalloprotease (*nas-9*), one NADH oxidase (*F17A9.5*) and one lectin (*clec-4*). Expression data was only available for *nas-9*, which is expressed in the hypodermis. The targets preferentially rescued in CY262 functioned in a variety of processes including stress resistance (3 glutathione S-transferases, 3 glucuronosyltransferases) and metabolism (1 fatty acid desaturase and 2 lipases). Expression data is available for 10 of the CY262-rescued targets. Eight of these targets are expressed intestinally, with 5 expressed exclusively in the intestine (*fat-5* fatty acid desaturase, *F09C8.1* phospholipase, *F54F3.3* lipase, *F42A10.6* unknown function and *lec-6* lectin). We note that the proportion of intestinal genes was the highest among the CY262-rescued targets (8/10, 80%) and was lower in the group of targets rescued in both CY251 and CY262 animals (5/11, 45%). We propose that the targets preferentially rescued in CY262 animals represent *age-1* targets that are autonomously regulated by intestinal *age-1* activity.

### *age-1* non-autonomously regulates a subset of stress response genes

Adult longevity and stress resistance in *daf-2* and *age-1* mutants results, at least in part, from transcriptional upregulation of stress-response genes [27], [31], [32]. Therefore, we examined our microarray data to determine whether the stress-resistance genes regulated by the *daf-2* and *age-1* pathway were autonomously or non-autonomously regulated (Table S3). Interestingly, *sod-3* expression was not rescued in CY262 or CY251 animals, suggesting this target is regulated cell-autonomously and is not under endocrine control. The heat shock genes targeted by heat shock factor, HSF-1, are major contributors to *daf-2* longevity [27], [28], [29], [33]. Our array results revealed that overexpression of *hsp-16* genes and *hsp-17* was rescued in CY262 and CY251, suggesting that small heat-shock proteins may be non-autonomous *age-1* targets. We note that, although the *hsp-16* genes were robustly upregulated in one *age-1(mg44)* sample, the were less robustly upregulated in an *mg44* replicate sample, so that the average change was not statistically significant. Nevertheless, these findings indicate that a subset of the stress response genes induced in *age-1* animals are non-autonomously regulated by *age-1*.

### Expression of an endocrine *age-1* target, *cyp-35B1*, is *daf-16* and *hsf-1*-dependent

One of the putative endocrine targets of *age-1* that we identified was *cyp-35B1*, which had previously been described as a *daf-2* pathway target named *dod-13* [30]. The *cyp-35B1* transcript was 17.8-fold overexpressed in *age-1(mg44)* hermaphrodites compared to wildtype and was rescued in both CY262 and CY251 (1.64-fold and 2.73-fold overexpressed vs wildtype, respectively). The *cyp-35B1* gene encodes a cytochrome P450 enzyme implicated in xenobiotic detoxification [34]. The *cyp-35B1* mRNA declines with age in wildtype hermaphrodites and *cyp-35B1* is a target of the *elt-3* GATA transcription factor that initiates and maintains intestinal cell fates in *C. elegans* [34].

**Figure 2:**
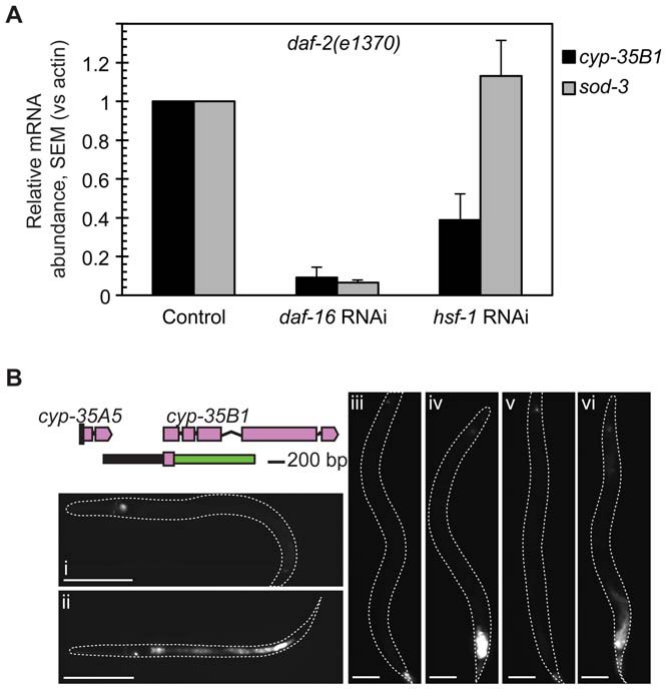
Regulation of *cyp-35B1* expression by *daf-16* and *hsf-1* in *daf-2(e1370)*. A. RT-PCR analysis of endogenous *cyp-35B1* and *sod-3* mRNA levels in *daf-2(e1370)* adults under control conditions or under *daf-16* or *hsf-1* RNAi. Results are average from three independent experiments. T-test was used to determine significance. *cyp-35B1* mRNA was significantly reduced by *daf-16* RNAi and *hsf-1* RNAi (*p* = 0.003 and 0.046, respectively). *sod-3* mRNA was significantly reduced by *daf-16* RNAi (*p*<0.001) but not by *hsf-1* RNAi (*p* = 0.55). B. Expression of *cyp-35B1:gfp* transcriptional reporter was low or undetectable in wildtype non-dauer larvae (*i*) and adults (*iii*), but was expressed in the intestine of dauer larvae (*ii*) and *daf-2(e1370)* adults (*iv*). In *daf-2(e1370)* adults, *cyp-35B1:*GFP expression was abrogated by *daf-16* RNAi (*v*) and substantially reduced by *hsf-1* RNAi (*vi*). The spot of GFP fluorescence in the head reflects the transformation marker, *gcy-7:*GFP, expressed in the ASE/L neuron. Bars, 100 µm. In some lines, *hsf-1* RNAi resulted in increased or broader intestinal *cyp-35B1*:GFP expression. Since this phenotype was not recapitulated by the endogenous *cyp-35B1* mRNA (panel A), we attribute this observation to mysregulation of the *cyp-35B1:gfp* transgene under conditions of reduced *hsf-1* activity.

Our microarrays showed that the *age-1*-dependent endocrine pathway could rescue *hsp-16* overexpression in *age-1* mutants. This observation led us to hypothesize that the *hsf-1* heat-shock transcription factor which regulates *hsp-16* expression and can collaborate with *daf-16*, might be important for expression of other endocrine *age-1* targets [28],[29]. We therefore examined whether *hsf-1* RNAi was also required for *cyp-35B1* expression in response to lowered *age-1* activity. Using semi-quantitative RT-PCR, we examined transcript levels for *cyp-35B1* and *sod-3* in *daf-2(e1370)* adult hermaphrodites treated with *daf-16* or *hsf-1* RNAi or an empty vector RNAi control (Fig. 2A). As expected, *sod-3* overexpression in *daf-2(1370)* adults was suppressed by *daf-16* RNAi, consistent with previous evidence showing that *sod-3* is a direct DAF-16 target [17]. Treatment with *daf-16* RNAi also reduced *cyp-35B1* mRNA in *daf-2(e1370)*, consistent with the identification of *cyp-35B1* as a target of the *daf-2* and *age-1* pathway ([30]; this work). In contrast, only *cyp-35B1* mRNA was significantly reduced in *daf-2(e1370)* animals treated with *hsf-1* RNAi, while *sod-3* levels were unaffected. This result demonstrates that *hsf-1* activity is required for induction of *cyp-35B1*, but not *sod-3*, in the absence of *daf-2* and *age-1* activity.

We next examined the tissue distribution of *cyp-35B1* by constructing a transcriptional reporter expressing GFP from a *cyp-35B1* promoter. A previously-described *cyp-35B1:gfp* reporter was expressed in an *elt-3*-dependent manner, suggesting the possibility of intestinal expression [34]. We identified the *cyp-35B1* promoter as a 660-bp sequence between the *cyp-35B1* initiating methionine and the next upstream gene, *cyp-35A5* (Fig. 2B). During reproductive development and in wildtype adults, *cyp-35B1:*GFP was either not detectable or expressed at very low levels in posterior hypodermal cells (Fig. 2B*i*, *iii*). In dauers and *daf-2(e1370)* adults, *cyp-35B1:*GFP was upregulated and expressed solely in the intestine (Fig. 2B*ii*, *iv*). Intestinal *cyp-35B1*:GFP levels in *daf-2(e1370)* adults were substantially reduced by *daf-16* RNAi (Fig. 2B*v*). Treatment with *hsf-1* RNAi also reduced levels of *cyp-35B1*:GFP in *daf-2(e1370)* adults, although not to the same extent as for *daf-16* RNAi (Fig. 2B*vi*). These findings together demonstrate that *cyp-35B1* expression under conditions of low *daf-2* pathway activity is regulated by coordinate action of *daf-16* and *hsf-1*. In some lines, we observed increased or broader intestinal expression of *cyp-35B1*:GFP in *hsf-1* RNAi-treated animals. We attribute these changes to misregulation of the GFP reporter transgene, as we never observed increased levels of the endogenous *cyp-35B1* mRNA under *hsf-1* RNAi conditions.

### Direct regulation of intestinal *cyp-35B1* expression by DAF-16 and HSF-1

**Figure 3:**
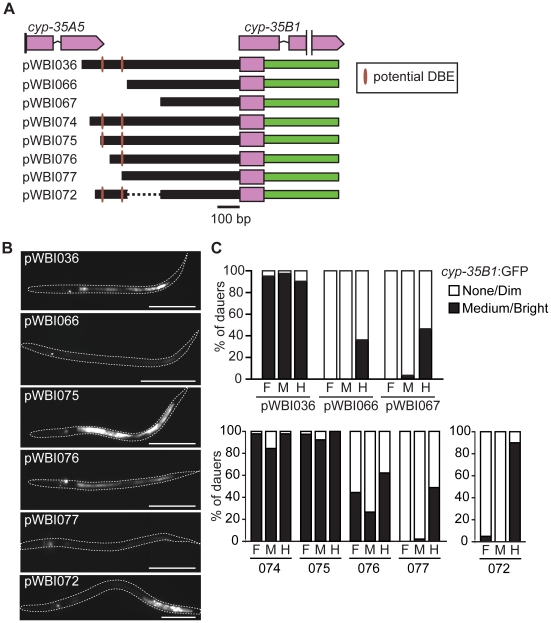
Identification of *cis*-regulatory modules (CRM) for dauer *cyp-35B1:gfp* expression by deletion analysis. Deletions in the *cyp-35B1* promoter were constructed to identify *cis*-regulatory modules necessary for directing dauer-specific expression in the intestine. (A) Diagram of *cyp-35B1* promoter constructs; promoters are black bars, brown hatches designate potential DAF-16 binding elements (DBE, [17]), pink boxes designate *cyp-35B1* exon 1 sequence fused to GFP (green bar). (B) Representative fluorescence images of *cyp-35B1*:GFP expression in dauer larvae carrying transgenes described in panel (A); bar, 100 µm. (C) GFP expression in dauer fore-, mid- and hindgut from indicated transgenes; black bars indicate % of dauers with medium or bright GFP fluorescence. GFP expression was scored in 20–59 dauers for each construct.

Based on our results, we hypothesized that *cyp-35B1* may be a direct or indirect target of DAF-16 and/or HSF-1. To test these possibilities, we studied the *cyp-35B1* promoter by deletion using *gfp* reporters. Deletion of 150-bp from the 5′ end of the 0.6-kb *cyp-35B1* promoter abolished intestinal GFP in dauers, without significantly affecting the weak hindgut expression observed in developing larvae (Fig. 3). Inspection of the deleted region identified two sequences (TTAAACA & AAAACA) resembling the previously-identified DAF-16 binding element (DBE, GTAAAC/TA) [17]. This suggested that *cyp-35B1* might be a direct DAF-16 target. Smaller deletions were made from this 150-bp region removing one or both of the DBEs. Deletion of sequences containing one DBE reduced, but did not eliminate, *cyp-35B1:*GFP in dauers (pWBI076), while deletion of both DBEs eliminated dauer expression (pWBI077). The DBE-containing region was not sufficient for intestinal *cyp-35B1:*GFP expression in dauers, however. In addition, maximal *cyp35B1:*GFP expression required an adjacent 100-bp sequence which lacked any DBE-like sequences (pWBI072).

**Figure 4:**
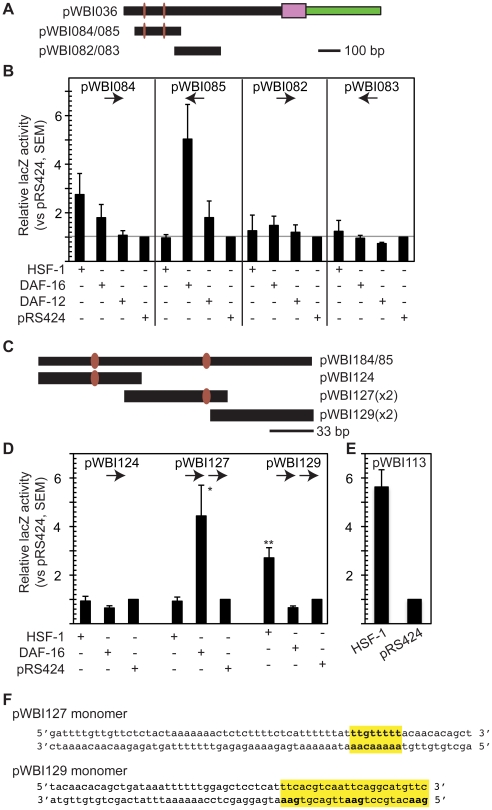
Yeast 1-hybrid analysis to detect DAF-16 and HSF-1 binding to *cyp-35B1* dauer CRM. Yeast 1-hybrid assays were performed to detect DAF-16 and HSF-1 binding to *cyp-35B1* dauer CRM (A, B) and subfragments (C, D). DAF-12 binding was also examined for the *cyp-35B1* dauer CRM (A, B), but produced negative results. (E) As a positive control, HSF-1 could bind to the heat-shock element (HSE)-containing region in the *hsp-16* promoter in pWBI113. In DAF-16- or HSF-1-expressing cells, transcription factor binding to the promoter fragments was measured as beta-galactosidase reporter activity in yeast cell extracts, normalized for cell density. Charts show average beta-galactosidase activity, relative to vector (pRS424) controls. Number of trials: pWBI082, pWBI083, 4 trials; pWBI084, 9 trials; pWBI085, 8 trials; pWBI124, 6 trials; pWBI127, 5 trials; pWBI129, 6 trials; pWBI113, 2 trials. Statistical significance was determined by t-test; *p = 0.05; **p = 0.01. Arrows under plasmid names depict the number and orientation of the *cyp-35B1* promoter fragments within the 1-hybrid reporter constructs. (F) Sequences of the *cyp-35B1* promoter fragments within pWBI127 and pWBI129. Highlighted sequence in pWBI127 fragment depicts a possible DAF-16 binding site; highlighted sequence in pWBI129 fragment show possible HSF binding site containing imperfect inverted repeats of 5′-AGAAN-3″ sequences.

To test whether DAF-16 and/or HSF-1 directly bind to the promoter regions shown to be required for *cyp-35B1:*GFP expression in dauers, we utilized the yeast 1-hybrid assay for DNA-protein interactions [35], [36], [37], [38]. We first constructed yeast expression plasmids for the two preys we wished to test, DAF-16 and HSF-1, utilizing a copper-inducible yeast expression system to drive expression of full-length *daf-16* or *hsf-1* cDNAs [39], [40]. Next, we constructed bait plasmids by inserting the 150-bp dauer CRM or a dispensable downstream region in front of a minimal promoter driving ß-galactosidase [40]. Four bait plasmids were tested, containing either the dauer CRM or the downstream region in the sense or antisense orientation with respect to the ß-galactosidase reporter (pWBI084/085 and pWBI082/083, respectively) (Fig. 4A). Reporter expression was activated by HSF-1 in yeast cells containing pWBI084, which contains the dauer CRM in the sense orientation with respect to the ß-galactosidase reporter. In the opposite orientation (pWBI085), ß-galactosidase expression was enhanced in cell expressing DAF-16. In contrast, we did not detect any stimulation of ß-galactosidase expression by the DAF-16 or HSF-1 preys in cells containing baits with promoter fragments that were dispensible for dauer *cyp-35B1:gfp* expression (pWBI082 or 083).

To further delineate the binding regions for DAF-16 and HSF-1 binding within the promoter fragments in pWBI084/085, we subdivided this 160-bp fragment into three overlapping fragments of 60–65-bp each. In yeast expressing DAF-16, ß-galactosidase expression was stimulated in the presence of the pWBI127 bait plasmid, which contains the *cyp-35B1* promoter fragment with the 2^nd^ DBE (Fig. 4C, D). The adjacent promoter fragment, carried in pWBI129, stimulated beta-galactosidase reporter expression in yeast expressing HSF-1. HSF transcription factors bind as trimers to the heat shock element (HSE) 5′-AGAANNTTCTAGAAN-3′, consisting of three inverted repeats of the 5′-AGAAN-3′ monomeric sequence. Inspection of the *cyp-35B1* promoter element contained in pWBI129 identified a sequence on the bottom strand which resembles this consensus (5′-GAAcaTgCctGAAttgaCgtGAA-3′), but may be an imperfect inverted pentamer repeat of the 5′-AGAAN-3′ sequence (Fig. 4F). The presence of an HSE-like sequence in pWBI129 is consistent with the observation of HSF-1 binding in the 1-hybrid assay.

## Discussion

The goal of this study was to identify potential endocrine targets of *age-1* activity that could be regulated non-autonomously from the nervous system and/or the intestine. Expression of wildtype *age-1* within neurons or intestinal cells rescues dauer arrest and lifespan phenotypes of *age-1(mg44)* animals [7], [8]. This evidence led to a working model whereby *age-1* activity within signaling tissues regulates an endocrine output that, in turn, can direct dauer morphogenesis and aging in target tissues [8]. The major genetic target of *age-1* is *daf-16*, encoding a FOXO transcription factor [15]. All available evidence indicates that the major mechanism for AGE-1/PI3K regulation of DAF-16 is cell-autonomous, via phosphorylation by AKT kinases regulated by AGE-1/PI3K phospholipid products [41], [42], [43]. Thus, the finding that *daf-16* acts cell-autonomously in the intestine to promote longevity was an apparent contradiction to earlier mosaic and transgenic analyses showing that the upstream regulators, *daf-2* and *age-1*, regulate these processes non-autonomously [6]. To resolve this conflict, we proposed that *age-1* can regulate *daf-16* activity in target tissues through convergent cell-autonomous and non-autonomous mechanisms [8].

To investigate possible effectors through which *age-1* might non-cell autonomously regulate *daf-16*, we used transcriptional microarrays to identify *age-1* target genes that could be non-autonomously regulated by *age-1*. This search identified 127 potential non-autonomous *age-1* target genes. We found that one of these, *cyp-35B1/dod-13*, which is expressed in the intestine of dauer larvae and *daf-2(e1370)* adults, could be directly regulated by both HSF-1 and DAF-16. Furthermore, other HSF-1 target genes, the *hsp-16* genes, were non-autonomously regulated by *age-1*. Expression of a subset of *daf-2* target genes is known to be *hsf-1*-dependent, although HSF-1 has not been shown to directly regulate these targets [28].

**Figure 5:**
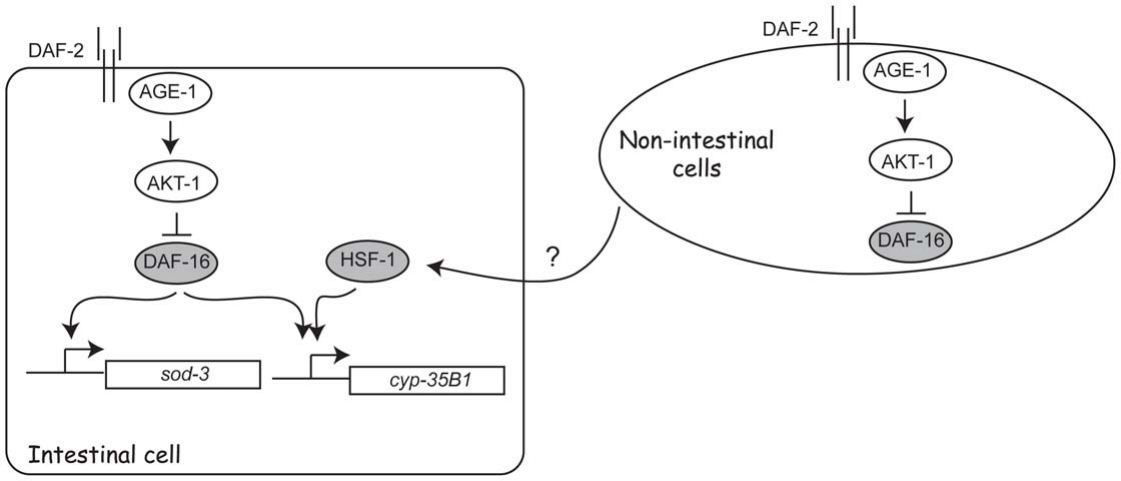
Regulation of intestinal *cyp-35B1/dod-13* expression by DAF-16 and HSF-1. Two intestinal DAF-16 target genes, *sod-3* and *cyp-35B1/dod-13*, are differentially regulated by HSF-1. Yeast 1-hybrid and promoter deletion mapping analyses suggest that DAF-16 and HSF-1 directly bind the *cyp-35B1* promoter. Quantitation of mRNA showed that *hsf-1* activity was required for *cyp-35B1* induction in dauers, but *hsf-1* was dispensible for *sod-3* induction. Microarray data indicated that *cyp-35B1* expression in *age-1* mutants could be regulated non-autonomously by *age-1* expression in neuronal cells (CY251), as well as cell-autonomously by *age-1* expression in the intestine (CY262). Thus, *cyp-35B1* is a possible endocrine target for *age-1* activity in neuronal cells. We propose that the *age-1* pathway may non-autonomously couple to *hsf-1* activity in intestinal cells to regulate *cyp-35B1* and other targets.

These observations provide circumstantial evidence placing *hsf-1* in the pathway for *daf-2* and *age-1* non-autonomy. We propose that DAF-2/IR and AGE-1/PI3K signaling in non-intestinal cells regulates an endocrine output that affects HSF-1 activity in intestinal cells (Fig. 5). We hypothesize that HSF-1 and DAF-16 can interact within intestinal cells to optimize expression of HSF-1 and DAF-16 target genes that may extend lifespan. An alternative model is that HSF-1 and DAF-16 function independently to promote expression of prolongevity target genes in intestinal cells. Distinguishing between these possibilities will likely require characterization of other non-autonomous *age-1* targets and clearer descriptions of the interactions between *hsf-1* and *daf-16* in the regulation of *C. elegans* dauer arrest and longevity.

## Materials and Methods

### Strains and growth

*C. elegans* strains were maintained at 15°C on NG agar medium with nystatin and streptomycin with OP50 bacteria as a food source [44]. The following strains were used: Bristol N2 (wildtype); SP75 (*sqt-1(sc13) age-1(mg44)/mnC1*); CY251 (*sqt-1(sc13) age-1(mg44)*; *bvIs2*); CY262 (*sqt-1(sc13) age-1(mg44)*; *bvIs1*); CY312 (*daf-16(mgDf50)*; *daf-2(e1370)*); DR40 (*daf-1(m40)*). *bvIs1* and *bvIs2* were previously described [8].

### Microarray analysis

Gene expression in CY262 and CY251 was compared to non-transgenic *age-1(mg44) m+z-*adults of similar age, and all comparisons were in reference to wildtype young adults. Synchronized populations of animals at the late-L4/young-adult stage were obtained from embryos isolated by bleach treating gravid hermaphrodites. Embryos were hatched overnight in S medium without food, causing developmental arrest at the first larval (L1) stage. Arrested L1s were plated with food, grown for 72 hours at 20°C and washed in M9. Total RNA was isolated with Absolutely RNA miniprep kit (Stratagene, La Jolla, CA). cDNA was labeled with the Quick Amp 2 Color Labeling Kit (Agilent Technologies, Santa Clara, CA) and hybridized to Agilent 2×22 k oligo microarrays containing probes for nearly the complete *C. elegans* genome. Slides were scanned using the Agilent Microarray Scanner (G2565B). Three biological replicates were examined per strain. After hybridization, probes with raw fluorescence signals >100 in wildtype samples were selected for further analysis as being reliable signals. Expression ratios for each gene were calculated in all 3 strains with respect to wildtype controls at similar, or slightly later developmental stage. Expression ratios were calculated as z-scores for a statistical evaluation of relative expression, and as fold-changes for standardized displays of relative expression [45]. To determine fold change, raw fluorescence values were first normalized by dividing the fluorescence intensity for a given sample by the average intensity of all samples for the array. Fold change ratios were then determined by dividing mean normalized value for all replicates of test versus control conditions. Statistical significance was judged using t-test to compare signal intensity measurements among biological replicates. Two replicates were performed for *age-1(mg44)* and 3 for CY251, CY262 and N2. MIAME-compliant raw microarray data is available from the NCBI Gene Expression Omnibus (Accession #GSE18200).

### *cyp-35B1:gfp* reporter

The *cyp-35B1* promoter was considered to be the sequences between the predicted translational start to the immediate upstream gene and was PCR-amplified from *C. elegans* genomic DNA using primers with unique restriction sites. Primer sequences were 5′-CAACAGAGGAGACAATGCCG-3′ and 5′-GGAAGAGAAACAGGTCCTGGTGGG-3′. PCR products were purified, digested and ligated into predigested pPD95.75, which contains GFP and the *unc-54* 3′UTR (Addgene, Cambridge, MA). Constructs were confirmed by DNA sequencing. Transgenic animals were created by microinjection transformation (100 ng/µl plasmid DNA with 50 ng/µl of the co-injection marker *gcy-7:gfp*). Stable transmitting lines were selected in the 2nd generation and analyzed.

For *cyp-35B1* promoter deletions, 150-bp and 300-bp deletions were made in the *cyp-35B1* promoter in pWBI036, to create pWBI066 and pWBI067, respectively, by PCR using upstream primers that hybridized to internal sites within the cloned promoter. The PCR primers also contained unique BamHI and KpnI restriction sites, which were used to insert the PCR fragments into pPD95.75. To further define the region responsible for dauer expression, smaller deletions were made from the region deleted in pWBI066, using the same PCR strategy, to make pWBI074 (59-bp deletion), pWBI075 (109-bp deletion), pWBI076 (159-bp deletion) and pWBI077 (209-bp deletion). Next, a 161-bp fragment containing both putative DAF-16 binding sites was PCR amplified with primers containing unique BamHI and NheI restriction sites and the digested product was ligated to a basal promoter in pWBI067, consisting of 398-bp upstream from the translational start, creating pWBI072.

GFP expression was compared in temperature-induced *daf-2(e1370)* dauers, starvation-induced wildtype dauers and *daf-2(e1370)* adults raised at 15°C and then held at 25°C for 24 hours. For microscopy, animals were either mounted on a 2% agar pad with levamisole and photographed with a Hamamatsu CCD camera (Hamamatsu Photonics, Japan) and OpenLab software (Improvision Inc., USA) on a Nikon E900 microscope (Nikon Corporation, Japan) or transferred onto an NGM plate spread with 20 µL of 10% sodium azide and photographed on a Nikon SMZ1500 stereodissecting microscope using a SPOT RT3 Slider camera and SPOT Advanced software (Diagnostic Instruments, USA). All images for a particular GFP reporter were collected using identical exposure times.

For RNAi experiments, populations were obtained from synchronized egg lay on plates seeded with dsRNA-expressing bacteria or control bacteria. Larvae were raised for at 25°C (4 days) for dauers, or 15°C (5 days) for adults that were subsequently transferred to 25°C for 24 hours before examination.

### Yeast 1-hybrid assays

Plasmids for yeast 1-hybrid assays were kindly provided by K. Yamamoto (UCSF). pYSYE0002 is a prey plasmid containing the DAF-12 N500 gene expressed from the copper inducible *cup-1* promoter and pYSYR0002 is a bait plasmid contain a DAF-12 responsive element cloned upstream of P*cyc-1* driving lacZ expression [40]. To construct the *daf-16* and *hsf-1* prey vectors, the *daf-16* or *hsf-1* cDNAs were amplified with primers containing Xma I and Not I restriction sites. PCR fragments were cloned into corresponding restriction sites in the pYSYE0002, and the resulting expression clones, pWBI109 and pWBI108, were sequence confirmed. Bait plasmids were constructed containing *cyp-35B1* promoter fragments were constructed using PCR-amplified promoter fragments with added NotI restriction sites. These PCR products were inserted in the place of the DAF-12 responsive element in pYSYR0002 and the resulting plasmids were sequence confirmed. Yeast were transformed with both a bait and prey plasmid, or negative controls, using the Yeastmaker Yeast Transformer Kit (Clontech, USA) with selection on SC medium lack tryptophan and uracil. For ß-galactosidase assays, independent yeast colonies were inoculated into 3-mL of selective medium and grown overnight at 30°C. Prey protein expression was induced by inoculation with 100 µM CuSO4 for 4–5 hours at 30°C. ß-galactosidase activity was assayed from pelleted cells using the Yeast ß-Galactosidase Assay Kit (Thermo Scientific, USA). The parent vector for pYSYE0002 is pRS424, which was used as a negative control for bait auto-induction.

## Supporting Information

Table S1 mRNA expression levels of *daf-2* pathway class 1 and 2 target genes in *age-1* mutants with tissue-restricted *age-1* expression. Expression levels for class 1 and 2 *daf-2* pathway targets (Murphy et al. 2003) were obtained from microarray data for *age-1(mg44)*, CY262 and CY251 relative to wildtype. Relative expression levels are presented as fold-changed relative to wildtype. “Missing from array?”, genes which were absent from the microarrays used in this study; “Our results”, indicates whether previous target classifications were consistent in the current study; “Rescue category”, indicates whether expression was rescued to wildtype or near-wildtype levels in CY262 and/or CY251 for targets that were congruent between the prior and current study; “Not rescued” indicates targets that maintained the mutant level of expression in CY262 and CY251; “Both” indicates targets whose expression was rescued to wildtype or near-wildtype levels in both CY251 and CY262.
(XLS)

Table S2 mRNA and anatomical expression data for genes upregulated by ≥2-fold in *age-1(mg44)* adults relative to wildtype. Expression data, as fold-change relative to wildtype and *age-1(mg44)*, is shown for each strain (*age-1(mg44)*, CY262 and CY251). “262 rescue”, “251 rescue” and “Category” indicate whether upregulation in *age-1(mg44)* was determined to be rescued in CY262 or CY251 and the corresponding rescue category (262, 251, Both, None). Anatomical expression data were obtained from the curated expression pattern annotations for each gene in WormBase (www.wormbase.org).
(XLS)

Table S3 10.1371/journal.pone.0017369.s003
Expression of stress response genes in *age-1(mg44)*, CY262 and CY251. mRNA expression levels were obtained from microarray data for genes annotated as heat-shock protein, glutathione S-transferase, catalase, superoxide dismutase, lysozyme or metallothionein. Expression levels in *age-1(mg44)*, CY262 and CY251 are shown as fold-change relative to wildtype.
(XLS)
